# Vitamin K1 prevents diabetic cataract by inhibiting lens aldose reductase 2 (ALR2) activity

**DOI:** 10.1038/s41598-019-51059-2

**Published:** 2019-10-11

**Authors:** R. Thiagarajan, M. K. N. Sai Varsha, V. Srinivasan, R. Ravichandran, K. Saraboji

**Affiliations:** 1 School of Chemical & Biotechnology, SASTRA University, Tamil Nadu, India; 2 Department of Biotechnology, Indian Institute of Technology, Madras, Chennai 600036 India; 3 Disease Program Lead – Diabetes, MedGenome Inc., Bangalore, India; 4 Diabetes Research Program, Division of Endocrinology, Department of Medicine, NYU Langone Medical Center, New York, NY 10016 USA; 5 Present Address: Department of Advanced Zoology & Biotechnology, Ramakrishna Mission Vivekananda College, Mylapore, Chennai 600004 India

**Keywords:** Vision disorders, Lens diseases

## Abstract

This study investigated the potential of vitamin K1 as a novel lens aldose reductase inhibitor in a streptozotocin-induced diabetic cataract model. A single, intraperitoneal injection of streptozotocin (STZ) (35 mg/kg) resulted in hyperglycemia, activation of lens aldose reductase 2 (ALR2) and accumulation of sorbitol in eye lens which could have contributed to diabetic cataract formation. However, when diabetic rats were treated with vitamin K1 (5 mg/kg, sc, twice a week) it resulted in lowering of blood glucose and inhibition of lens aldose reductase activity because of which there was a corresponding decrease in lens sorbitol accumulation. These results suggest that vitamin K1 is a potent inhibitor of lens aldose reductase enzyme and we made an attempt to understand the nature of this inhibition using crude lens homogenate as well as recombinant human aldose reductase enzyme. Our results from protein docking and spectrofluorimetric analyses clearly show that vitamin K1 is a potent inhibitor of ALR2 and this inhibition is primarily mediated by the blockage of DL-glyceraldehyde binding to ALR2. At the same time docking also suggests that vitamin K1 overlaps at the NADPH binding site of ALR2, which probably shows that vitamin K1 could possibly bind both these sites in the enzyme. Another deduction that we can derive from the experiments performed with pure protein is that ALR2 has three levels of affinity, first for NADPH, second for vitamin K1 and third for the substrate DL-glyceraldehyde. This was evident based on the dose-dependency experiments performed with both NADPH and DL-glyceraldehyde. Overall, our study shows the potential of vitamin K1 as an ALR2 inhibitor which primarily blocks enzyme activity by inhibiting substrate interaction of the enzyme. Further structural studies are needed to fully comprehend the exact nature of binding and inhibition of ALR2 by vitamin K1 that could open up possibilities of its therapeutic application.

## Introduction

Aldose reductase inhibition is one of the best strategies to inhibit polyol pathway and its associated complications in diabetes. Sorbitol is the first product of the pathway and is further broken down to fructose and fructose by itself leads to production of free radicals. One major consequence of sorbitol formation is that it can accumulate within lens cells resulting in osmotic stress^1^. It is thus clear that sorbitol and fructose formation could lead to the breakdown of normal lens physiology, triggering cataract formation. A lot of studies thus have suggested that the inhibitors of aldose reductase could have the ability to limit certain diabetic complications^2–4^. Many aldose reductase inhibitors (ARI), both synthetic and natural, have been tested in animal models. Some of the natural ARIs include, ethyl acetate extract from leaf of *Aegle marmelos*^5^, glucogallin from *Embilica officinalis*^6^, flower bud extract from *Magnolia fargesii*^7^, roots of *Pueraria lobata*^8^, extracts of *Litchi chinensis*^9^, curcumin^10^, *Zingiber officinale*^11,12^, while some of the synthetic ARIs include zopolrestat^13–16^, tolrestat^17–19^, sorbinil^20^ and fidarestat^21^. However, though synthetic ARIs have gained prominence their efficacy in terms of ALR inhibition in diabetic complications, especially in neuropathy, has not been satisfactory^22–25^. Our focus is to identify natural compounds with potential ARI properties. In our recent study, preliminary findings suggested that vitamin K1 could possess potent lens ARI activity and this was also supported by a reduction in lens sorbitol accumulation^26^. To further explore the potential of vitamin K1 as an effective ARI we compared it with zopolrestat, a standard ARI, and tried to understand the mechanism of action of AR inhibiting activity of vitamin K1.

Zopolrestat is an acetic acid derivative and is known to inhibit AR activity, thereby controlling the level of sorbitol, fructose and myo-ionositol in lens, retina, kidney and sciatic nerves of diabetic rats^27^. Studies with zopolrestat have been performed in normal and diabetic rats^28^ and healthy male volunteers^29^ and it has shown 85% absorption^30^ without major side effects. Though there are reports on increased half-life of zopolrestat in certain tissues like nerve, eye and kidney^28^, it has been shown to be relatively safe and also partially improved functional abnormalities in left ventricles of diabetic patients^31^. Nevertheless, a more recent report by Bouguerra *et al*.^32^ has demonstrated eryptosis triggered by zopolrestat at relevant *in vivo* dosages, which could be an important point to consider during its usage.

As far as vitamin K is concerned, studies have shown the importance of its members in controlling both diabetes and its associated complications. In diabetes, vitamin K2 has been shown to be important for acute insulin response^33^, insulin sensitivity and modulating glycemic status^34–36^, prevention of bone loss in diabetic patients^37^, blocking insulin resistance^38^, inhibiting production of inflammatory cytokines associated with insulin response^39^ and modulating dephospho uncarboxylated protein^40^.

Even though the exact mechanism as to how vitamin K1 improves insulin sensitivity and reduces glycemic status in type 2 diabetes remains unclear, studies have proposed that osteocalcin plays a crucial role in glucose metabolism by increasing the secretion of insulin, enhancing β islet cell proliferation and by increasing the expression of adiponectin in adipocytes, thereby increasing insulin sensitivity^41–43^. Studies have also shown that when men with lower phylloquinone were tested for oral glucose tolerance there was decreased insulin and an increase in the glucose level when compared to men with higher phylloquinone level that showed an increase in insulin secretion and decreased blood glucose^43,44^. More recently, studies on isolated mouse islets have shown that menaquinone-4 potentiated glucose induced insulin secretion suggesting an insulinotrophic potential for vitamin K family^45^. Indeed, one previous study has also shown that circulating levels of vitamin K1 is inversely proportional to risk of type 2 diabetes in humans^46^.

In addition to these, our studies with vitamin K1 have shown that vitamin K1 could prevent hyperglycemia by protecting pancreatic islets^47^ and thus inhibit type 1 diabetes-induced cataractogenesis^26^. In addition to this, if vitamin K1 could prove to be an ARI, then this would be a unique compound unlike other ARI, wherein it has two important activities, (1) anti-hyperglycemic and (2) ARI. Thus, in this study we probed the mechanisms of aldose reductase inhibition by vitamin K1 and compared it with a synthetic aldose reductase inhibitor zopolrestat.

## Materials and Methods

Streptozotocin, β-NADH, thiobarbituric acid (TBA), sorbitol dehydrogenase and DL- glyceraldehyde were procured from Sigma Aldrich. Zopolrestat was gifted by Dr. Ravichandran Ramasamy, NYU Langone Medical Center, New York and human recombinant aldose reductase (BioVision, Catalog No: 7361-100, ALR2) was a generous gift from Dr. Srinivasan Vedantham, SASTRA University.

### Experimental animals

Male albino Wistar rats (200–225 g) were procured from Central Animal Facility, SASTRA University, Thanjavur. All experiments were approved by Institutional Animal Ethics Committee (IAEC), Central Animal Facility, SASTRA University (125/SASTRA/IAEC/RPP). All experiments were carried out in accordance with CPCSEA (Committee for the Purpose of Control and Supervision of Experiments on Animals) guidelines and regulations. Rats were housed in an air-conditioned room (22 ± 2 °C) with a lighting schedule of 12 h light: 12 h dark and fed with commercial rat diet from Provimi Animal Nutrition, India, Pvt. Ltd.

### Diabetes induction, vitamin K1 dosage, blood and lens collection

Animals were acclimatized to laboratory conditions for 1 wk. STZ was administered after 4 h of fasting as a single, intraperitoneal injection (35 mg/kg) in sterile citrate buffer (0.1 M; pH 4.5)^26,47^. After 72 h, blood from tail vein was withdrawn and glucose estimated using Microdot glucometer. Rats with glucose levels < 220 mg/dL were excluded from the experiment and the remaining rats were randomized into two groups of seven animals each.

Group 1: control

Group 2: STZ

Group 3: STZ plus vitamin K1. Vitamin K1 (5 mg/kg body weight, subcutaneous) was administered 72 h after STZ injection twice a week for 3 months. Vitamin K1 is not toxic and the dosage used here is based on our previous studies^26,47^.

Group 4: STZ plus zopolrestat. Zopolrestat (25 mg/kg body weight, oral gavage^13^,) was administered 72 h after STZ injection twice a week for 3 months.

Body weight was measured at regular intervals.

### Preparation of lens homogenate

At the end of the study, animals were sacrificed by carbon dioxide inhalation. Eye lens was immediately dissected and washed in ice-cold saline. About 100 mg of the eye lens was taken and homogenized in tris-HCl buffer (0.1 M; pH 7.4) at 4 °C to obtain a 10% homogenate. The lens homogenate was centrifuged at 11,000 × g for 30 min and the supernatant obtained was stored at −70 °C until its use for further biochemical analysis. All the assays were carried out within 48 hours after sample collection.

### Estimation of lens aldose reductase and sorbitol in STZ-induced diabetic rats

Sorbitol in lens was estimated as per our previous report^26^. Lens aldose reductase (ALR2) activity was estimated according to^47^. Briefly, in a test tube the following were mixed together; 100 mL each of 50 mM potassium phosphate buffer (pH 6.2), 0.4 M lithium sulphate, 5 mm 2-mercaptoethanol, and 10 mM of DL-glyceraldehyde. After incubation (37 °C, 2 min), reaction was initiated with the addition of 100 mL of 0.1 mM NADPH and 100 mL lens tissue homogenate (enzyme source). Optical density was measured at 340 nm and AR activity expressed as nmol/min/mg protein.

### Nature of inhibition of lens aldose reductase enzyme by vitamin K1

In this, we made an attempt to understand the nature of ALR2 inhibition by vitamin K1 under various experimental conditions.

#### Effect of vitamin K1 pre- and co-incubation on control lens ALR2 activity

100 µl of the control eye lens homogenate was incubated first with 1.5 mg of vitamin K1 for 5 min. 1.5 mg is the average dosage of vitamin K1 administered to each rat when measured at a concentration of 5 mg/kg bw. After incubation, 100 µl of 50 mM potassium phosphate buffer (pH 6.2), 100 µl of 0.4 M lithium sulphate, 100 µl of 5 mM 2-mercaptoethanol and 100 µl of 10 mM of DL-glyceraldehyde was added and further incubated at 37 °C for 2 min and the reaction was initiated with the addition of 100 µl of 0.1 mM NADPH. Optical density was measured at 340 nm against a blank containing buffer instead of the homogenate. ALR2 activity was expressed as nmoles of NADPH oxidized/min/mg protein. For co-incubation studies, 100 µl of the control eye lens homogenate was incubated for 5 min with 1.5 mg of vitamin K1 along with reagents for ALR2 measurement as mentioned above. At the end of incubation, the reaction was initiated with the addition of 100 µl of 0.1 mM NADPH and ALR activity measured as described above.

#### Effect of time of pre-incubation of vitamin K1 on control lens ALR2 activity

100 µl of control eye lens homogenate was pre-incubated with 1.5 mg of vitamin K1 for different time points (0, 5, 10, 15, 20, 25, and 30 min). At the end of incubation, lens ALR2 activity was measured as mentioned above.

#### Effect of vitamin K1 concentration on control lens ALR2 activity

100 µl of control eye lens homogenate was pre-incubated with different concentrations of vitamin K1 (0.0664 fM, 33.20 pM, 0.74 nM, 3.32 mM, and 166 M) and incubated for 5 min. At the end of incubation, lens ALR2 activity was measured as mentioned above.

### Nature of inhibition of recombinant human aldose reductase (ALR2) by vitamin K1

#### Effect of NADPH concentration on recombinant human aldose reductase activity pre-incubated with vitamin K1

1 µg of recombinant human aldose reductase was pre-incubated for 5 min with 0.74 nM vitamin K1. After incubation, 50 µl each of 50 mM potassium phosphate buffer (pH 6.2), 0.4 M lithium sulphate, 5 mM 2-mercaptoethanol and 10 mM DL-glyceraldehyde were added and incubated at 37 °C for 2 min. The reaction was initiated with the addition of 50 µl of NADPH (0.01, 0.05, 0.1, 0.5 or 0.5 mM). ALR activity was measured as described above.

#### Effect of DL-glyceraldehyde concentration on recombinant human aldose reductase activity in the presence or absence of vitamin K1

1 µg of recombinant human aldose reductase was pre-incubated with 0.74 nM of vitamin K1 for 5 min or the recombinant human ALR was added directly to a reaction mixture (without vitamin K1) containing 50 µl each of 50 mM potassium phosphate buffer (pH 6.2), 0.4 M lithium sulphate, 5 mM 2-mercaptoethanol and DL-glyceraldehyde (5, 10, 15, 20, 25 or 30 mM) and incubated at 37 °C for 2 min. ALR activity was measured as mentioned above.

#### Effect of vitamin K1 concentration on recombinant human aldose reductase activity

1 µg of recombinant human aldose reductase was incubated for 5 min with vitamin K1 (0.0664 fM, 33.20 pM, 0.74 nM, 3.32 mM, or 166 M). After incubation, ALR activity was measured as mentioned above.

#### Effect of zopolrestat on recombinant human aldose reductase activity

1 µg of recombinant human aldose reductase was incubated for 5 min with zopolrestat (0.74, 72, 144, 216 or 719 nM). Zopolrestat concentration was based on the work of Nakano and Petrash^48^. After incubation, ALR activity was measured as mentioned above.

### In silico analysis

#### Molecular structure

To perform molecular docking between aldose reductase (ALR2)/aldehyde reductase (ALR1) and vitamin K1, three dimensional structures were obtained from RCSB Protein Data Bank (accession ids: 4IGS and 2ALR, for ALR2 and ALR1, respectively), which are solved at 0.85 Å and 2.48 Å resolution, respectively^49,50^. Further, protein preparation wizard from Schrödinger was used to remove non-protein atoms, water molecules from the PDB files and to correct the structural defects in raw state such as missing atoms and residues^51^. All the visualizations of molecular structures were performed with PyMOL (Schrödinger, LLC)^52^. The chemical structure of vitamin K1 was drawn using ACD/Chemsketch and saved in mol2 file format. LigPrep was used from Schrödinger software to take 2D structures and produce the corresponding low-energy 3D structures with correct chiralities^53^. Minimization was performed using OPLS_2005 force field and Epik ionizer^54^ at the standard pH of 7 and with maximum number of conformers per structure as 1000 with RMSD 1.0 Å.

#### Molecular docking

In the present study, we have used Glide software (Grid-based Ligand Docking with Energetics), available in Schrödinger suite^55^, for the molecular docking studies. Glide uses a hierarchical series of filters to search for possible locations of the ligand in the active-site region of the receptor. Further, it exhibits excellent docking accuracy and high enrichment across a diverse range of receptor types. Since the active site of ALR1/ALR2 are highly conserved, based on various co-factors bound ALR structures, the binding site residues are identified which includes Thr19, Trp20, Lys21, Asp43, Tyr48, His110, Ser159, Asn160, Ser210, Ser214, Lys262 and Asn272^56^. Further, receptor grid generation program^56^ was used to define the volume of grid box set to centroid of the active site residues and the number of docking runs was set as 1000. In the present study, we have used the Extra precision (XP) scoring function for molecular docking, as it uses only good ligand poses to perform docking and it gives better result than the SP (standard-precision) scoring function^57^. The predicted protein ligand complexes were optimized and ranked according to the empirical docking scoring function, calculated using the following equation:

1 $${\rm{GScore}}={\rm{0.065}}\ast {\rm{vdW}}+{\rm{0.130}}\ast {\rm{Coul}}+{\rm{Lipo}}+{\rm{Hbond}}+{\rm{Metal}}+{\rm{BuryP}}+{\rm{RotB}}+{\rm{Site}}$$

where, vdW-van der Waals energy, Coul - Coulomb energy, Lipo- Lipophilic contact term, HBond - hydrogen-bonding term, Metal- metal-binding term, BuryP - penalty for buried, polar groups, RotB - penalty for freezing rotatable bonds, Site- polar interactions at the active site and the coefficients of vdW and Coul are a = 0.065, b = 0.1.

### Molecular dynamics simulations and protein-ligand binding free energy calculation

Molecular dynamics (MD) simulations for the docked complexes of ALR2 and ALR1 bound with Vitamin K1 were performed using GROMACS 5.1.2 package^58^ with charmm27 force field^59^ for all amino acid residues. The ligand topologies were generated using SwissParam online server^60^. All the MD simulations were done at 300 K for 50 ns at the in-house facility, 11 TF High-Performance Computing cluster. The protein was completely solvated in a cubic box containing TIP3P water model^61^ and for setting up the MD system for simulation we followed the same protocols as used in our earlier work^62^. The protein-ligand binding free energy (ΔG) was calculated from the MD trajectories using the software program myPresto version 5 (https://www.mypresto5.jp/en/)^63^.

### Spectrofluorimetry analysis

Spectrofluorimetric analysis was performed to analyze the change in the secondary structure of the recombinant human ALR2 due to binding of vitamin K1, DL-glyceraldehyde, NADPH or zopolrestat. The reaction mixture consisted of varying combinations of 30 µl each of human recombinant ALR2, vitamin K1, DL-glyceraldehyde, NADPH or zopolrestat, made up to 150 µl with 50 mM potassium phosphate buffer (pH 6.2) so that the final concentration was 1 µg/ml of recombinant human ALR2, 0.74 nM of vitamin K1, 10 mM of DL-glyceraldehyde, 0.1 mM of NADPH and 0.74 nM of zopolrestat. The fluorescence was obtained by exciting at 280 nm and scanned between 300–500 nm in a Jasco spectrofluorometer FP- 8200.

### Protein estimation

Protein from the eye lens homogenate was estimated according to Lowry *et al*.^64^.

### Statistical analysis

All enzyme assays and spectrofluorometry studies were performed in duplicates. Results are expressed as mean ± S.D of at least seven determinations. One-way analysis of variance (*p* < 0.05) was followed by Tukey’s post hoc test to evaluate the significant difference between STZ + vit K1, STZ and between STZ + zopolrestat, STZ.

## Result

### Effect of vitamin K1 and zopolrestat on blood glucose

**Figure 1:**
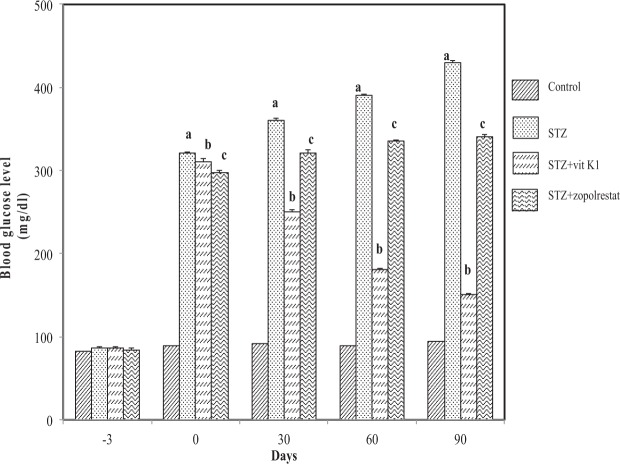
Effect of vitamin K1 and zopolrestat on blood glucose. Values are mean ± SD of 5 determinations. Blood was collected from all treatment groups every month and processed for glucose measurement. ‘a’ indicates the difference observed between groups STZ and control and ‘b’ indicates the difference observed between STZ + vit K1 and STZ, ‘c’ indicates the difference between STZ + zopolrestat and STZ. All differences are statistically significant at *P* < 0.05.

As shown in Fig. 1, STZ-administration lead to an increase in blood glucose level when measured after 3^rd^ day and constantly increased as seen at the end of first month (360.25 ± 12.25 mg/dL, *p* < 0.05) and till the end of the study at 90 days (430 ± 8.5 mg/dL, *p* < 0.05). Whereas in STZ + vit K1 group, due to vitamin K1 administration there was a gradual decrease in blood glucose level at the end of first month (250 ± 9.87 mg/dL, *p* < 0.05) till the end of the study at 90 days (150.21 ± 6.78 mg/dL, *p* < 0.05). However, as expected in STZ + zopolrestat group, the blood glucose level at the end of the first month were observed to be increased (320.89 ± 9.21 mg/dL, p < 0.05) when compared to control (92 ± 5.21 mg/dL) and blood glucose levels remained high till the end of the study at 90 days (340.25 ± 7.89 mg/dL, p < 0.05). We would like to point out that the observed glucose levels in the case of zopolrestat treated diabetic rats was nevertheless still lower than STZ alone administered (group II) rats, at the end of 90 days. This has not been demonstrated with previous reports^65^ on zopolrestat and we do not have a clear explanation other than speculate on the strain of animal used. However we would like to emphasise here that in this study animals with blood glucose more than 220 mg/dL were considered to be diabetic/hyperglycemic and thus this also falls into that category, notwithstanding the lower levels when compared to STZ alone rats. These results suggest that vitamin K1 had more beneficial effect in terms of decrease in blood glucose level in diabetic rats, when compared to zopolrestat and is very effective in inhibiting hyperglycemia, which could contribute to its anti-diabetic function.

### Both vitamin K1 and zopolrestat inhibit lens aldose reductase (ALR2) activity and sorbitol accumulation

**Figure 2:**
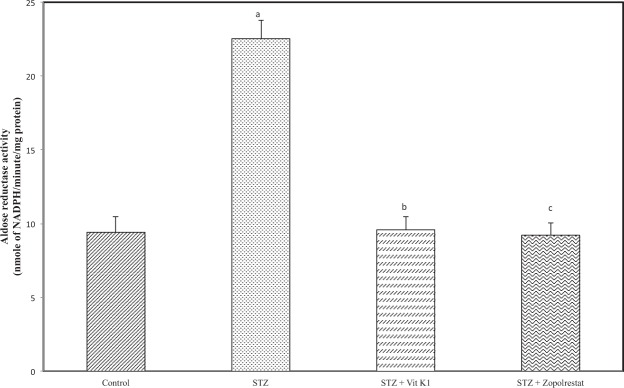
Effect of vitamin K1 and zopolrestat on lens aldose reductase activity in diabetic lens. Values are mean ± SD of 5 determinations. ‘a’ indicates the difference observed between groups STZ and control and ‘b’ indicates the difference observed between STZ + vit K1 and STZ, ‘c’ indicates the difference between STZ + zopolrestat and STZ. All differences are statistically significant at *P* < 0.05.

**Figure 3:**
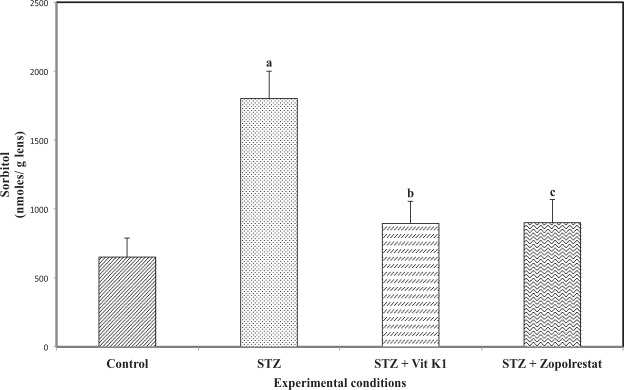
: Effect of vitamin K1 and zopolrestat on lens sorbitol. Values are mean ± SD of 5 determinations. ‘a’ indicates the difference observed between groups STZ and control and ‘b’ indicates the difference observed between STZ + vit K1 and STZ, ‘c’ indicates the difference between STZ + zopolrestat and STZ. All differences are statistically significant at *P* < 0.05.

Activation of polyol pathway is a major factor in diabetic complications especially in diabetic cataract. Aldose reductase is the first and rate limiting enzyme of the polyol pathway to be activated by hyperglycemia and we compared the ALR2 inhibiting potential of vitamin K1 with zopolrestat. The activity of aldose reductase (Fig. 2) was increased significantly in STZ – induced diabetic rats (22.53 ± 1.21 nmoles of NADPH oxidized/min/mg protein, *p* < 0.05) due to hyperglycemia. As expected treatment of diabetic rats with zopolrestat, a known inhibitor of ALR2, resulted in a significant inhibition of lens ALR2 activity (9.21 ± 0.85 nmoles of NADPH oxidised/min/mg protein, *p* < 0.05), when compared to untreated diabetic rats. It should be mentioned here that we got an inhibition of around 59% with zopolrestat whereas in one of the earlier studies, the authors have shown greater inhibition at much lower concentrations of zopolrestat^66^. This could be attributed to differences in experimental conditions. However, when compared to STZ group, there was a significant reduction. Similarly, treatment of diabetic rats with vitamin K1 also resulted in a significant inhibition of lens ALR2 activity (9.58 ± 0.89 nmoles of NADPH oxidized/min/mg protein, *p* < 0.05) when compared to untreated diabetic rats. As shown in Fig. 3, increase in ALR2 activity due to hyperglycemia resulted in an increase in the level of sorbitol (1801.21 ± 199.25 nmoles/g lens, *p* < 0.05) in STZ-induced rats as compared with the controls (650.32 ± 136.21 nmoles/ g lens). As expected, treatment of diabetic rats with zopolrestat (900.21 ± 167.21 nmoles/ g lens, *p < *0.05) or with vitamin K1 (895.23 ± 136.21 nmoles/ g lens) resulted in significant decrease in sorbitol accumulation, when compared to untreated diabetic rats. These results suggest that by inhibiting ALR2, both zopolrestat and vitamin K1 were able to inhibit sorbitol accumulation in diabetic lens.

### Nature of inhibition of control lens aldose reductase (ALR2) activity by vitamin K1

**Figure 4:**
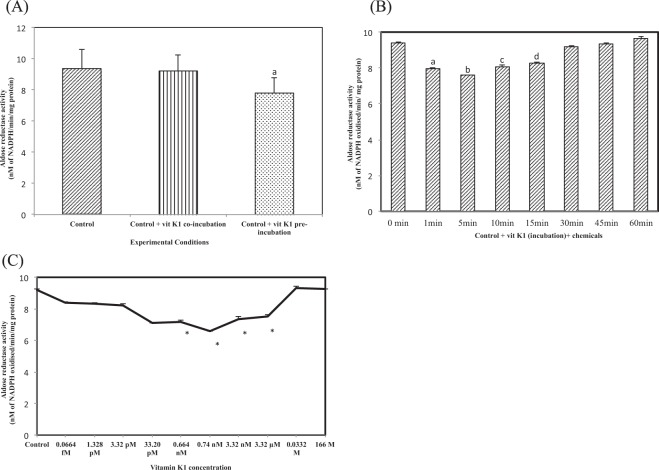
(**A**) Competitive inhibition of aldose reductase activity by vit K1 in control eye lens. Values are mean ± SD of 5 determinations. ‘a’ indicates the difference observed between control + vit K1 + chemicals and control + vit K1 preincubation + chemicals. (**B**) Inhibition of aldose reductase activity by vit K1 in control eye lens: Effect of time of pre-incubation of vitamin K1. ‘a’ indicates the difference observed between control and control + vit K1 pre-incubation; ‘b’ indicates the difference observed between 1 min pre-incubaton and no incubation; ‘c’ indicates the difference observed between 5 min pre-incubation and no incubation; ‘d’ indicates the difference observed between 10 min pre-incubation and no incubation and ‘e’ indicates the difference observed between 15 min incubation and no incubation and these are statistically significant at *P* < 0.05 (**C**). Effect of vitamin K1 concentration on control lens aldose reductase activity. Values are mean ± SD of 3 determinations. ‘*’represents the difference between vitamin K1 and control are statistically significant at *P* < 0.05.

(i) In the first of these experiments, we made an attempt to understand the effect of pre- or co-incubation of vitamin K1 on lens ALR2 activity. As shown in Fig. 4A, co–incubation of lens homogenate with vitamin K1 resulted in a slight but non-significant (9.21 ± 1.05 nM of NADPH oxidized/min/mg protein) inhibition in lens ALR2 activity. The values were similar to control lens (9.36 ± 1.25 nM of NADPH oxidized/min/mg protein) ALR2 activity. However, when vitamin K1 was pre-incubated with lens homogenate, there was a stronger and significant inhibition of lens ALR2 activity (7.8 ± 0.98 nM of NADPH oxidized/min/mg protein, *p* < 0.05).

(ii) Next, we analyzed the effect of pre–incubation time of vitamin K1 with lens homogenate on lens ALR2 activity. For this, vitamin K1 was incubated with lens homogenate for 1, 5, 10, 15, 30, 45 and 60 min and then ALR2 activity was assayed. As shown in Fig. 4B, with an increase in pre-incubation time up to 15 min, there was a corresponding inhibition of lens ALR2 activity, but maximum inhibition was observed at 5 min (7.6 ± 0.02 nM of NADPH oxidized/min/mg protein, *p* < 0.05), when compared to control (9.4 ± 0.04 nM of NADPH oxidized/min/mg protein). At 10 min (8.07 ± 0.08 nM of NADPH oxidized/min/mg protein, *p* < 0.05) and 15 min (8.29 ± 0.08 nM of NADPH oxidized/min/mg protein, *p* < 0.05), the activity was lower than control but higher than that obtained at 5 min pre–incubation. Beyond 15 min, increase in pre-incubation time resulted in rescue of lens ALR2 activity and the activity at 60 min pre-incubation (9.36 ± 0.06 nM NADPH oxidized/min/mg protein) was similar to control lens ALR2 activity.

(iii) Next, we determined the effect of increase in vitamin K1 concentration on lens aldose reductase activity. For this, we tested vitamin K1 at the following concentrations: 0.0664 fM, 1.328 pM, 3.32 pM, 33.20 pM, 0.664 nM, 3.32 nM, 3.32 µM, 0.0332 M and 166 M. In addition, we also included 0.74 nM, which is the actual concentration of vitamin K1 measured in two lenses from diabetic animals that were treated with 5 mg/kg bw vitamin K1 twice a week for 90 days. Control lens were pre-incubated with vitamin K1 for 5 min and then ALR2 activity was assayed. As can be seen in Fig. 4C increase in vitamin K1 concentration up to 3.32 µM (7.54 ± 0.09 nM of NADPH oxidized/min/mg protein, *p* < 0.05) resulted in significant inhibition in lens ALR2 activity when compared to control lens. However, maximum inhibition was observed at 0.74 nM (6.59 ± 0.02 nM of NADPH oxidized/min/mg protein, *p* < 0.05) and this was followed by 33.2 pM (7.1 ± 0.05 nM of NADPH oxidized/min/mg protein, *p* < 0.05) and 0.664 nM (7.19 ± 0.07 nM of NADPH oxidized/min/mg protein, *p* < 0.05). At very high concentrations, vitamin K1 failed to inhibit control lens ALR2 activity (0.0332 M = 9.3 ± 0.054 nM of NADPH oxidized/min/mg protein; 166 M = 9.26 ± 0.08 nM of NADPH oxidized/min/mg protein). Hence, 0.74 nM appears to be the optimum concentration required to produce maximum inhibition of lens ALR2 activity. Thus, for all further studies the following parameters were used: vitamin K1 concentration = 0.74 nM and incubation time = 5 min.

### Nature of inhibition of recombinant human aldose redcutase (ALR2) activity by vitamin K1

Having established the potential of vitamin K1 in inhibiting ALR2 activity in control lens homogenate, we made an attempt to understand the effectiveness of viatmin K1 in interacting with and inhibiting recombinant human aldose reductase enzyme (ALR2) to validate our findings with lens homogenate.

**Figure 5:**
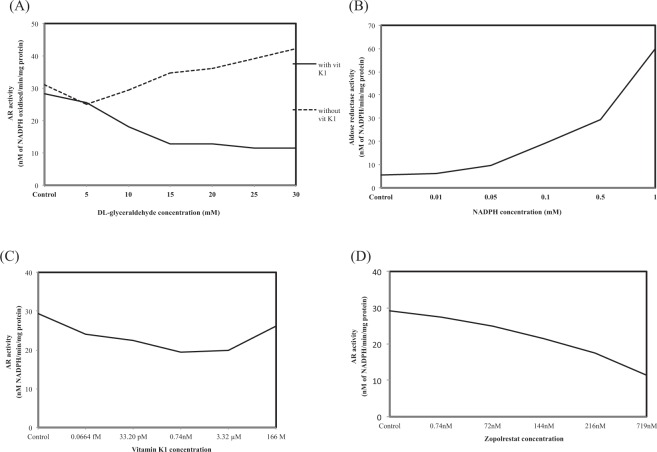
(**A**) Effect of DL-glyceraldehyde concentration on recombinant human aldose reductase activity in the presence or absence of vitamin K1. Values are mean of 2 determinations. (**B**) Effect of NADPH concentration on recombinant human aldose reductase activity in the presence of vitamin K1. Values are mean of 2 determinations. (**C**) Effect of vitamin K1 concentration on recombinant human aldose reductase activity. Values are mean of 2 determinations. (**D**) Effect of zopolrestat concentration on recombinant human aldose reductase activity. Values are mean of 2 determinations.

(i) To determine whether an increase in substarte concentration could rescue recombinant human ALR2 activity inhibition by vitamin K1, we analysed ALR2 activity in the presence of increasing concentrations of DL-glyceraldehyde (5, 10, 15, 20, 25 and 30 mM) and in the presence or absence of 0.74 nM of vitamin K1. As shown in Fig. 5A, when concentration of DL-glyceraldehyde was increased from 5 mM (25.03 nM of NADPH oxidized/min/mg protein) to 30 mM (42.13 of NADPH oxidized/min/mg protein), there was a corresponding increase in the activity of recombinant human aldose reductase enzyme in the absence of vitamin K1. However, there was a significant decrease in recombinant human ALR2 activity in the presence of vitamin K1 and even an increase in substrate concentration failed to rescue this inhibition by vitamin K1.

(ii) Next we determined whether increase in the concentration of co-factor NADPH, could rescue recombinant human ALR2 activity. For this, human ALR2 was incubated with different concentrations of NADPH (0, 0.01, 0.05, 0.1, 0.5 and 1 mM) in the presence or absence of 0.74 nM vitamin K1. As shown in Fig. 5B, in controls, (i.e) without NADPH or vitamin K1, the human ALR2 showed an activity of 5.53 nM of NADPH oxidized/min/mg protein. However, when NADPH concentration was increased there was a corresponding increase in the activity of human ALR2 and the activity was not inhibited in the presecnce of vitamin K1. This suggests that at saturating concentrations, NADPH could reverse ALR2 inhibition by vitamin K1.

(iii) To check the effect of increase in vitamin K1 concentration on recombinant human ALR2 activity, the enzyme was incubated for 5 min with different concentrations of vitamin K1 (0, 0.0664 fM, 33.21 pM, 0.74 nM, 3.32 µM and 166 M). The reaction contained 10 mM of DL-glyceraldehyde and 0.1 mM of NADPH. As shown in Fig. 5C, increase in vitamin K1 concentrations produced a significant inhibition of recombinant human ALR2 activity with 0.74 nM producing the maximum inhibition (19.32 nM of NADPH oxidized/min/mg protein) when compared to control (29.31 nM of NADPH oxidized/min/mg protein). 3.32 µM of vitamin K1 also produced a significant inhibition of recombinant human ALR2 activity (19.92 nM of NADPH oxidized/min/mg protein) but the value was nearer to that of 0.74 nM. However, when concentration of vitamin K1 was increased to 166 M, there was no inhibition (26.20 nM of NADPH oxidized/min/mg protein) in ALR2 activity. This pattern of inhibition was quite similar to that observed with crude preparations of control lens homogenate.

(iv) To further validate the ALR2 inhibitory potential of vitamin K1, we estimated the activity of recombinant human ALR2 in the presence of different concentrations of zopolrestat, a well known ALR inhibitor. The concentrations used were 0, 0.74, 72, 144, 216 and 719 nM^28,29^. As shown in Fig. 5D, increase in the concentration of zopolrestat lead to a corresponding and significant decrease in recombinant human ALR activity with lowest activity seen at 719 nM of zopolrestat (11.47 nM of NADPH oxidized/min/mg protein), when compared to control recombinant human ALR2 activity (29.1 nM of NADPH oxidized/min/mg protein). These results are quite comparable with vitamin K1 and suggests the potential of vitamin K1 as a novel ALR2 inhibitor.

Taken together the results on the nature of inhibition of recombinant human ALR2 by vitamin K1, suggests that vitamin K1 has a very good potential in interacting with and subsequently inhibiting the aldose reductase enzyme. Together with the earlier findings on lens ALR2 activity, we show the potential of vitamin K1 as a novel and potent ALR inhibitor.

### *In silico* analysis of vitamin k1 binding in active site of ALR

The molecular simulation and docking was performed on human aldose reductase (ALR2) and aldehyde reductase (ALR1) molecules as described in the methods section. Though the amino acid sequence similarity between ALR1 and ALR2 is 50%, the overall three-dimensional structures are highly similar and the R.M.S. deviation between ALR1 and ALR2 was found as 0.76 Å. The binding mode of vitamin K1in the NADPH binding site is highly comparable in both ALR1 and ALR2 structures. Interestingly the present docking study clearly displays the location of vitamin K1, which partially overlaps the NADPH binding site and thus hinders the NADPH availability for ALR. The docking glide XP score for vitamin K1 with ALR1 and ALR2 were found to be −5.013 kcal/mol and −8.869 kcal/mol, respectively with corresponding glide Emodel scores of −50.1 kcal/mol and −75.8 kcal/mol. Emodel scores indicates the estimated conformational energy of the ligand. Further, Molecular dynamics simulation data for the docked conformations of ALR1 and ALR2 with vitamin K1 was generated for 50 ns. The compactness of the protein fold was ensured by monitoring time evolution of radius of gyration of the complexes, root-mean-square deviation (RMSD) and Root Mean Square Fluctuation (RMSF). Interestingly, we observed that the binding mode of vitamin K1 in ALR is consistent during the entire 50 ns simulation (Supplementary Fig. S1) and the protein (ALR2)-ligand (vitamin K1) binding free energy (ΔG) was in the range of −3.45 to −5.03 kcal/mol (Supplementary Fig. S2).

**Figure 6:**
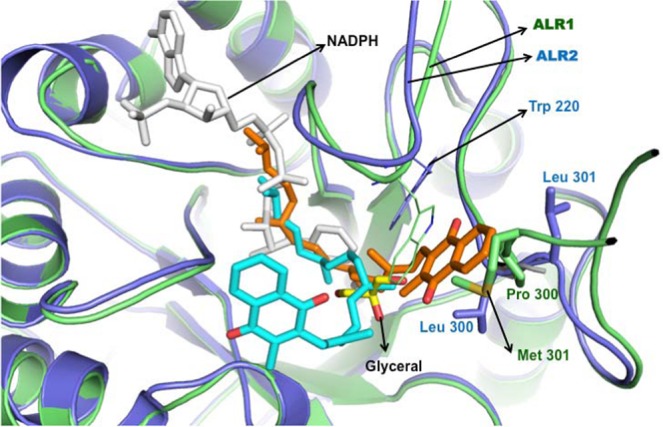
The structural superposition of ALR1 (green) and ALR2 (blue) protein structures in cartoon representation. The docked conformations of vitamin K1 in ALR1 (cyan sticks) and ALR2 (orange stickes) shows the competitive binding in NADPH binding site (white sticks). The binding site residues Trp 220, Pro 300 and Met 301 in ALR1 are shown in green sticks and Trp 220, Leu 300 and Leu 301 in ALR2 are shown in blue sticks. The glycerol molecule in its bnding site is shown in yellow sticks, which is not in overlap with NADPH site but locates in the center of vitamin K1 binding site.

**Figure 7:**
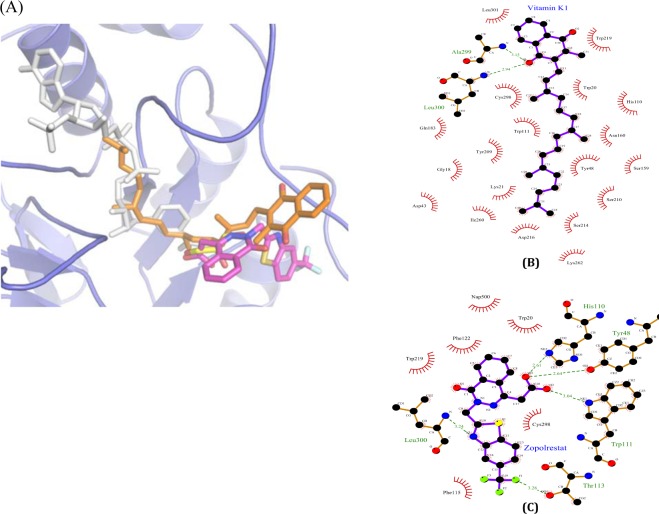
(**A**) Close-up view of docked conformation of vitamin K1 in ALR2 shows the overlap with the NADPH (white sticks, PDB id: 4IGS), Zopolrestat (magenta sticks, PDB id: 2HVO), and glyceraldehyde (yellow sticks, PDB id: 3V36) binding sites. 2D representation showing the interactions of vitamin K1 (**B**) and Zopolrestat (**C**) with ALR2 residues. The ligands and protein are shown with purple and brown bonds respectively. Hydrogen bond are shown in dotted lines along with donor-acceptor distance and residues interacting by hydrophobic interactions were represented as lines in red. The diagrams are prepared using LIGPLOT (Wallace *et al*., (1996). Protein Eng., 8, 127–134).

The larger deviation in vitamin K1 docked conformation found at the naphthoquinone moiety due to Leu300 and Leu301 residues, which facilitates hydrophobic interactions in ALR2; whereas in ALR1, naphthoquinone moiety is slightly reoriented due to the polar substitutions (Pro300, Met301) and the side chain conformation of Trp 220, which is sterically incompatible to the naphthoquinone moiety (Fig. 6). In contrast, the long hydrophobic tail of the vitamin K1 adopts highly similar conformation in both ALR1 and ALR2 in the binding site which comprises Trp 20, Tyr 209, Pro 211, Leu 212, Ile 260, Pro 261 that partially occupies the NADPH binding site. Figure 7(A) (also see Supplementary Fig. S3) shows the binding sites of zopolrestat, vitamin K1, NADPH and DL-glyceraldehyde in the ALR2 protein. It appears that vitamin K1 binds to both DL-glyceraldehyde and NADPH binding site of ALR2 and thus causes inhibition of the enzyme.

### Spectrofluorimetric analysis of aldose reductase activity

**Figure 8:**
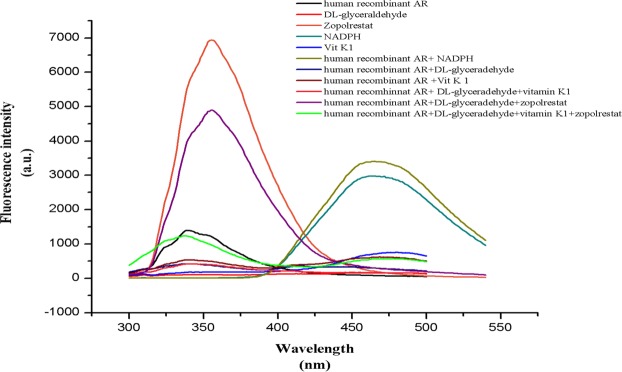
Spectrofluorimetric analysis of recombinant human aldose reductase.

Spectrofluorimetric analysis was perfromed inorder to confirm the binding of vitamin K1 in ALR active site. Spectra for recombinant aldose redcutase was performed by exciting at 280 nm and scanning from 300–500 nm (Fig. 8). An emission maxima was obtained at 339 nm (intensity 1397 a.u.) for the native protein whereas DL-glyceraldehyde, NADPH, vitamin K1 and zopolrestat shows an emission maxima at 341 nm (intensity 110 a.u.), 462 nm (intensity 2906 a.u.), 477 nm (intensity 750 a.u.) and 356 nm (intensity 6937 a.u.), respectively.

Interestingly, the complex of recombinant protein and DL-glyceradehyde produced an emission maxima at 339 nm; the complex of recombinant protein and vitamin K1 gave an emission maxima at 340 nm, which indicates the binding of DL-glyceraldehyde and vitamin K1 without altering the protein significantly. In contrast, the complex of recombinant protein with NADPH shows a shift in the emission maxima at 456 nm. This shift could be due to the alteration of structural re-arrangement that happens during the binding of NADPH in the buried region of protein molecule.

However, when the protein is complexed with both DL-glyceraldehyde and vitamin K1, the emission maxima was closer to 342 nm which is similar to emission spectra obtained for complex of protein and DL-glyceraldehyde/ protein and vitamin K1. Surprisingly, when zopolrestat was added along with the above complex, the emission maxima was shifted to 378 nm which could be due to the overlap in the binding site of zopolrestat and vitamin K1 which could lead to a structural change in the protein.

## Discussion

Aldose reductase mainly known for its role in catalyzing the first step in polyol pathway is also important in maintenance of osmotic homeostasis^67^, conversion of steroids^68^ and in reactive aldehyde detoxification^69,70^. Previous studies have shown that the inhbition of aldose reductase activity could limit the complications arising from diabetes^2,71,72^. In this paper we investigated the nature of ALR inhibition by vitamin K1 using lens homogenates from normal rats and recombinant human aldose reductase enzyme.

Zopolrestat, a well known synthetic ALR inhibitor, was used to compare and validate the ALR inhibitory potential of vitamin K1. STZ-administration to male Wistar rats resulted in diabetes with elevated blood glucose levels, while vitamin K1 treatment of diabetic rats resulted in significant reduction in blood glucose. However, treatment of diabetic rats with zopolrestat did not affect hyperglycemia as that compared to vitamin K1 treated animals.

Zopolrestat, a synthetic ALR inhibitor^4^, has been implicated for its anti-cataract potential. This is due to the fact that hyperglycemia is known to trigger lens aldose reductase enzyme^4^ leading to osmotic imbalance^73^ and cataractogenesis^73^. Thus, inhibiting ALR could potentially prevent diabetic complications such as cataract. In one earlier study, Beyer-Mears and Cruz^20^ have shown the anti-cataract potential of sorbinil, wherein treatment of diabetic rats with sorbinil helped to maintain lens transparency. Similarly zopolrestat has been shown to be an effective ALR inhibitor, however, there are issues concenrning its efficacy and toxicty.

Hyperglycemia-induced polyol pathway has been implicated in a variety of diabetic complications^72^ including cataract^4^. A previous study by Lee *et al*. have shown the involvement of aldose redcutase enzyme in a transgeneic mice over expressing ALR in eye lens^73^. Through this study some of the key roles of ALR in diabetic cataract formation has been established and more importantly validated. The role of ALR in mediating diabetic- and galactose-induced cataract, the ability of ALR in converting glucose to its polyol and the fact that polyol accumulation in the eye lens is one of major contributors for cataract formation has been established through this study. More importantly the same group have also shown in detail the involvement of ALR in diabetic cataract formation and the ability of sorbitol to bring about osmotic stress which is a key player in development of diabetic cataract^73^. Another study^74^ has shown that in transgeneic diabetic mice, expresing human ALR in the eye lens, developed sugar cataract whereas in wild type diabetic mice expressing very low ALR, it failed to develop cataract in the eye lens^75^. Thus, it is suggested that inhibition of aldose reductase enzyme, the first and rate limiting enzyme of the polyol pathway, could prove beneficial in terms of diabetic complications. Activation of polyol pathway leads to cellular damage primarily by sorbitol and fructose formation and accumulation^76^. It is well known that sorbitol-induced osmotic stress^73^ and fructose–induced free radical generation^77^ could possibly contribute to tissue damage, leading to cataract formation^71^. Though, both synthetic and natural ALR inhibitors are currently being tested^10,13^, these have issues associated with their efficacy and side effects. Moreover, the inhibitors of ALR do not work equally well with other isoforms of the enzyme^10^. In addition, there is the issue of plasticity of the active site of ALR^10,78^, which makes it difficult to design and develop potent ARIs. In our preliminary analyses, we showed that vitamin K1 was able to inhibit lens ALR2 and thus reduce lens sorbitol levels. This was further confirmed, when we compared vitamin K1 with zopolrestat for its ALR2 inhibitory potential. Treatment of diabetic rats with either vitamin K1 or zopolrestat resulted in significant inhibition of lens ALR2 activity and lens sorbitol accumulation. The level of inhibition by vitamin K1 was comparable with that of zopolrestat. This suggests that vitamin K1 could be a novel and potent ALR2 inhibitor and this ALR2 inhibitory potential could also contribute to its anti-cataract activity. It also leads us to speculate that this inhibition could possibly be due to a direct interaction of vitamin K1 and lens ALRs and we made an attempt to understand the nature of this interaction.

To further validate the ARI potential of vitamin K1 and to ascertain the possible interaction between vitamin K1 and ALRs, *in silico* studies were performed. The beneficial effect of ALR-inhibitors in preventing diabetic complications provides strong support to the hypothesis that ALR1 and ALR2 inhibition could be an effective strategy in the prevention or delay of diabetic cataract. However, several ALR inhibitor studies are inconsistent with observations found in experimental animals and also in clinical trials to assess efficacy against various diabetic complications^22,79,80^. Interestingly, our *in silico* docking analysis shows that vitamin K1 competitively binds in both ALR1 and ALR2 and this binding site overlaps ~50% of the NADPH binding site. The binding mode of vitamin K1 in both ALR1 and ALR2 is highly similar except the orientation of naphthoquinone moiety. In ALR2, the residues Leu300 and Leu301 stabilizes the binding of naphthoquinone moiety through hydrogen bonding and hydrophobic interactions; whereas in ALR1 the replacements Pro300 and Met301 is sterically incompatible to naphthoquinone moiety and flipped it to the other direction to interact with Ile49, Phe50, Phe125, Ala219, Trp220 (Figs 6 and 7B). However, interestingly the hydrophobic tail of the vitamin K1 adopts similar binding conformation in core of both ALR1 and ALR2 by forming hydrophobic interactions with Trp20, Tyr48, His110, Asn160, Tyr209, Pro211, Leu212, Ser214, Trp 111, Trp219, Ile260 and Cys298 that partially occupies the NADPH binding site (Fig. 7A,B). Of these the residues Tyr48, His110 and Trp111 were involved in stabilizing the conformation of zopolrestat (PDB id: 2HVO) by hydrogen bonding interactions (Fig. 7C). In comparison with zopolrestat, vitamin K1 was further stabilized with hydrogen bonding and hydrophilic interactions with Ala299 and Leu301 residues, respectively. This clearly suggests that due to the binding of vitamin K1 in the hydrophobic core, the binding site may not be available for NADPH and thus the enzyme activity is inhibited and production of sorbitol is reduced. This inhibition supports our experimental observation that presence of vitamin K1 suppresses the sorbitol accumulation under high glucose conditions.

This observation is in contrast to the earlier *in silico* studies that show that the reduced sorbitol production in only ALR2 is due to the non-competitive inhibition by curcumin (where curcumin fails to inhibit ALR1)^10^, whereas the present study shows the possibility of utilising vitamin K1 as a common inhibitor for both ALR1 and ALR2. Interestingly, the binding site residues are similar for both vitamin K1 and curcumin (Thr19, Trp20, Tyr48, His110, Trp111, Ile260, Leu300, Leu301), except the hydrophobic tail’s conformation of vitamin K1. This clearly indicates the importance of blocking the NADPH binding site to inhibit both ALR1 and ALR2, irrespective of the replacements found at Leu300 and Leu301.

To understand the nature of interaction and inhibition of lens ALR2 by vitamin K1, we performed a series of experiments under different conditions using lens homogenate from non-diabetic healthy animals. Results from vitamin K1 concentration in eye lens of vitamin K1 administered rats and dose-dependency suggested that 0.74 nM could be the optimum dosage for effective ALR2 inhibition by vitamin K1. It should be noted here that increase in vitamin K1 concentration, over and above 0.74 nM, failed to inhibit ALR2 activity over that observed with 0.74 nM. This we speculate could be due to the saturation of binding site between vitamin K1 and ALR2, which could have resulted in no significant change in ALR2 inhibition with increasing concentrations of vitamin K1. Use of vitamin K1 pre-incubation and co-incubation with control lens homogenate showed that vitamin K1 pre-incubation was more effective in inhibiting lens ALR2 activity. This suggests that prior presence of vitamin K1 is essential for it to interact effectively with the enzyme and produce inhibition. However, under co-incubation conditions, it is possible that the active sites are already occupied and vitamin K1 is unable to bind. Similarly, time for pre-incubation suggested that 5 min is the optimum. Even though inhibition of ALR2 was observed at 1, 10 and 15 min, maximum inhibition was observed at 5 min. Beyond 15 min vitamin K1 had no effect on ALR2 inhibition. The reason we speculate for no inhibition of ALR2 activity after 15 min of pre-incubation could be the loss of interaction between vitamin K1 and ALR2 after around 15 min of incubation. This we think is possible since the interaction between vitamin K1 and ALR2 is based on weak bonding. So with time this interaction becomes weaker and weaker resulting in the separation of vitamin K1 from the active site of the enzyme.

To further validate the inhibitory potential of vitamin K1, recombinant human aldose reductase (ALR2) was used in inhibition assays. The ALR2 assay was performed with various concentration of the substrate, DL-glyceraldehyde, in the presence or absence of 0.74 nM vitamin K1 and with 5 min pre-incubation. The results were surprising as the human ALR2 activity was inhibited with increase in the concentration of DL-glyceraldehyde and in the presence of vitamin K1. This is in contrast to the available literature where the ALR activity was generally found to increase with increasing concentration of the substrate in the presence of an inhibitor^10,22^. However, when the assay was performed in the absence of vitamin K1, there was an increase in the activity of ALR2 with increase in the concentration of the substrate. Since, there was a contradictory result in the activity of ALR2 with increase in concentration of DL-glyceradehyde, assays were performed by varying the concentration of NADPH which is the cofactor for ALR. The activity of ALR2 increased with increase in concentration of NADPH even when vitamin K1 was present and these results suggests that vitamin K1 probably interferes more with substrate binding at the active site of the enzyme than with co-factor binding.

The crystal structure of NADPH and glyceraldehyde bound form^49^ clearly shows that there is no overlap in their binding sites. Interestingly the docking studies show that the binding site of vitamin K1 overlaps with the binding position of glyceraldehyde. In order to verify this *in silico* observation, we have performed an ALR inhibitory assay using recombinant ALR2 with 0.1 mM NADPH, 0.74 nM of vitamin K1 and with varying concentration of DL-glyceraldehyde. The assay shows that in the absence of vitamin K1, the ALR2 activity increased with increase in concentration of DL-glyceraldehyde. However, in the presence of vitamin K1, the ALR2 activity showed a decreasing trend with increase in DL-glyceradehyde concentration. This effect may be due to the non-availability of binding site for glyceraldehyde upon binding of vitamin K1.The assay clearly confirms the possibility of having overlapping binding sites of glyceraldehyde and vitamin K1 which may hinder the role of glyceraldehyde.

A study by Muthenna *et al*.^10^ using curcumin as ARI demonstrated that curcumin at a concentration of 100 µM was effective in inhibiting sorbitol level in RBC under high glucose concentrations. In another study, two compounds orientin and isoorientin isolated from the leaves of *Colocasia esculenta* were used to explore their inhibitory potential on rat eye lens ALR. Orientin was used at a concentration of 1.65 µM and isoorientin at 1.92 µM^81^. Of all the concentrations of vitamin K1 assayed, 0.74 nM of vitamin K1 was effective in inhibitng ALR2 activity. Though there is a difference in the nature of these molecules and perhaps on the mechanism of their interaction with ALR, when compared to other studies vitamin K1 was able to inhibit the activity of ALR2 even at very low concentrations and this was further compared with zopolrestat. Zopolrestat dosage was selected based on Nakano and Petrash^48^. In their work they have shown through fluorescence study that zopolrestat and substrate, DL-glyceradehyde, bind at the same site leading to inhibition of ALR activity. In the present study, inhibition of ALR2 activity was observed with increasing concentartion of zopolrestat and the inhibition pattern was similar to the inhibitory activity of vitamin K1 on human ALR. Thus, these results show that the binding of vitamin K1 in the active site of human ALR is potential enough to inhibit its activity.

In order to confirm the binding of vitamin K1 in the active site of recombinant ALR2, spectrofluorimetry studies were performed. The complex of protein, substrate and the inhibitor molecules were analysed at the concentrations described in the methods section. The fluorescence intensity maxima of native protein, (without inhibitor or co-factor) and DL-glyceraldehyde was independently observed at 339–341 nm with an intensity of 1397 a.u. and 110 a.u., respectively. Whereas zopolrestat, NADPH and vitamin K1 gave an emission maxima at 356 nm (intensity 6937 a.u.), 462 nm (intensity 2906 a.u.) and 477 nm (intensity 750 a.u.), respectively. Recombinant protein along with cofactor NADPH was scanned for emission maxima. The emission maxima of the protein (339 nm) obtained is the excitation wavelength for NADPH and hence it transfers the fluorescence resonance energy to give an emission maxima at 465 nm with a fluorescence intensity of 3404 a.u. Due to high fluorescence intensity, the protein peak obtained at 339 nm was not significant.

Whereas when recombinant protein along with glyceraldehyde and along with vitamin K1 was scanned separately emission maxima was obtained at 339–342 nm, which is consistent with maxima observed when all the three are complexed (protein, glyceradehye and viatmin K1). Further, we repeated the fluorescence measurements with a standard inhibitor, zopolrestat by replacing vitamin K1 which shows similar fluorescence maxima. When both zopolrestat and vitamin K1 are complexed with recombinant ALR2 along with DL-glyceraldehyde, the fluorescence maxima shifts to 378 nm. This may be due to the overlapping binding site of vitamin K1 and zopolrestat as observed in the molecular docking studies (Fig. 7B,C).

Overall the fluorescence studies confirms the binding of vitamin K1 in the active site of recombinant ALR2 which is overlapping with the binding site of zopolrestat. These results also lead us to speculate that viatmin K1 binding to ALR2 active site is more on substarte binding site than co-factor binding site. However, it is interesting to note that vitamin K1 is able to occupy both these sites which are important for the optimal activity of the enzyme. Further, structural studies are needed to fully elucidate the interaction of vitamin K1 with lens ALR2. Nevertheless, these results conclusively show that vitamin K1 indeed interacts with ALR2 and inhibits it, thus demonstrating that vitamin K1 could be a novel and potent ARI with potential clinical applications.

## Supplementary information

Vitamin K1 prevents diabetic cataract by inhibiting lens aldose reductase 2 (ALR2) activity

## References

[1] Dvornik E (1973). Polyol accumulation in galactosemic and diabetic rats: control by an aldose reductase inhibitor. Science..

[2] Sestanj K (1984). N-[5-(trifluoromethyl)-6-methoxy-1-naphthalenyl-thioxomethyl]- N-methylglycine (Tolrestat), a potent, orally active aldose reductase inhibitor. J. Med. Chem..

[3] Kador PF, Robison WG, Kinoshita JH (1985). The pharmacology of aldose reductase inhibitors. Annu. Rev. Pharmacol. Toxicol..

[4] Banditelli S (1999). A new approach against sugar cataract through aldose reductase inhibitors. Exp. Eye. Res..

[5] Sankeshi V (2013). Inhibition of aldose reductase by Aegle marmelos and its protective role indiabetic cataract. J. Ethnopharmacol..

[6] Chang KC (2013). Beta-glucogallin reduces the expression of lipopolysaccharide-induced inflammatory markers by inhibition of aldose reductase in murine macrophages and ocular tissues. Chem. Biol. Interact..

[7] Lee Jun, Kim Nan Hee, Nam Joo Won, Lee Yun Mi, Jang Dae Sik, Kim Young Sook, Nam Sang Hae, Seo Eun-Kyoung, Yang Min Suk, Kim Jin Sook (2010). Scopoletin from the flower buds of Magnolia fargesii inhibits protein glycation, aldose reductase, and cataractogenesis Ex Vivo. Archives of Pharmacal Research.

[8] Kim NH, Kim YS, Lee YM, Jang DS, Kim JS (2010). Inhibition of aldose reductase and xylose-induced lens opacity by puerariafuran from the roots of Pueraria lobata. Biol. Pharm. Bull..

[9] Lee SJ, Park WH, Park SD, Moon HI (2009). Aldose reductase inhibitors from Litchi chinensis Sonn. J. Enzyme. Inhib. Med. Chem..

[10] Muthenna P, Suryanarayana P, Gunda SK, Petrash JM, Reddy GB (2009). Inhibition of aldose reductase by dietary antioxidant curcumin: Mechanism of inhibition, specificity and significance. FEBS. Lett..

[11] Kato A (2006). Inhibitory effects of Zingiber officinale Roscoe derived components on aldose reductase activity *in vitro* and *in vivo*. J. Agric. Food. Chem..

[12] Antony P, Vijayan R (2015). Identification of Novel Aldose Reductase Inhibitors from Spices: A Molecular Docking and Simulation Study. PLoS One..

[13] Badawy D, El-Bassossy HM, Fahmy A, Azhar A (2013). Aldose reductase inhibitors zopolrestat and ferulic acid alleviate hypertension associated with diabetes: effect on vascular reactivity. Can. J. Physiol. Pharmacol..

[14] Qiu Longxin, Lin Jianhui, Xu Fangui, Gao Yuehong, Zhang Cuilin, Liu Ying, Luo Yu, Yang James Y. (2012). Inhibition of Aldose Reductase Activates Hepatic Peroxisome Proliferator-Activated Receptor-αand Ameliorates Hepatosteatosis in Diabetic db/db Mice. Experimental Diabetes Research.

[15] Carvalho VF (2006). Aldose reductase inhibitor zopolrestat restores allergic hyporesponsiveness in alloxan-diabetic rats. Eur. J. Pharmacol..

[16] Dan Q (2004). Interaction between the polyol pathway and non-enzymatic glycation on mesangial cell gene expression. Nephron. Exp. Nephrol..

[17] Azal O (2002). Effects of aminoguanidine and tolrestat on the development of ocular and renal structural changes in experimental diabetic rats. Diabetes. Obes. Metab..

[18] Kador PF, Lee JW, Fujisawa S, Blessing K, Lou MF (2000). Relative importance of aldose reductase versus nonenzymatic glycosylation on sugar cataract formation in diabetic rats. J. Ocul. Pharmacol. Ther.

[19] Lee SH, Woo HG, Baik EJ, Moon CH (2000). High glucose enhances IL-1beta-induced cyclooxygenase-2 expression in rat vascular smooth muscle cells. Life. Sci..

[20] Beyer-Mears A, Cruz E (1979). Reversal of diabetic cataract by sorbinil, an aldose reductase inhibitor. Diabetes..

[21] Drel VR (2008). Aldose reductase inhibitor fidarestat counteracts diabetes-associated cataract formation, retinal oxidative-nitrosative stress, glial activation, and apoptosis. Int. J. Mol. Med..

[22] Pfeifer MA, Schumer MP, Gelber DA (1997). Aldose reductase inhibitors: the end of an era or the need for different trial designs. Diabetes..

[23] Raskin P, Rosenstock J (1987). Aldose reductase inhibitors and diabetic complications. Am. J. Med..

[24] Jaspan JB, Towle VL, Maselli R, Herold K (1986). Clinical studies with an aldose reductase inhibitor in the autonomic and somatic neuropathies of diabetes. Metabolism..

[25] Foppiano M, Lombardo G (1997). Worldwide pharmacovigilance systems and tolrestat withdrawal. Lancet..

[26] Sai Varsha MKN, Thiagarajan R, Manikandan R (2014). Inhibition of diabetic-cataract by vitamin K1 involves modulation of hyperglycemia-induced alterations to lens calcium homeostasis. Exp. Eye. Res..

[27] Schneider RP, Ericson JF, Lynch MJ, Fouda HG (1993). Confirmation of danofloxacin residues in chicken and cattle liver by microbore HPLC electrospray ionization tandem mass spectrometry. Biomed. Mass. Spectrom..

[28] Inskeep PB, Reed AE, Ronfeld RA (1991). Pharmacokinetics of zopolrestat, a carboxylic acid aldose reductase inhibitor, in normal and diabetic rats. Pharm. Res..

[29] Inskeep PB, Ronfeld RA, Peterson MJ, Gerber N (1994). Pharmacokinetics in man of the aldose reductase inhibitor zopolrestat. J. Clin. Pharmacol..

[30] Schneider RP, Fouda HG, Inskeep PB (1998). Tissue distribution and biotransformation of Zopolrestat, an aldose reductase inhibitor, in rats. Drug. Metab. Dispos..

[31] Johnson BF (2004). Cardiac abnormalities in diabetic patients with neuropathy: effects of aldose reductase inhibitor administration. Diabetes Care.

[32] Bouguerra G, Bissinger R, Abbès S, Lang F (2015). Zopolrestat induced suicidal death of human erythrocytes. Cell Physiol. Biochem..

[33] Seyama Y (2000). Comparative effects of vitamin K2 and estradiol on experimental arteriosclerosis with diabetes mellitus. Int. J. Vitam. Nutr. Res..

[34] Yoshida M, Jacques PF, Meigs JB (2008). Effect of vitamin K supplementation on insulin resistance in older men and women. Diabetes Care.

[35] Rees K, Guraewal S, Wong YL (2010). Is vitamin K consumption associated with cardio-metabolic disorders: a systematic review. Maturitas.

[36] Iwamoto J, Seki Sato Y, Matsumoto H, Takeda T, Yeh JK (2011). Vitamin K2 prevents hyperglycemia and cancerous osteopenia in rats with streptozotocin-induced type 1 diabetes. Calcif. Tissue Int..

[37] Uenishi K (2012). Diabetes mellitus and osteoporosis. Dietary therapy of diabetes related osteoporosis. Clin. Calcium.

[38] Yoshida M, Booth SL, Meigs JB, Saltzman E, Jacques PF (2008). Phylloquinone intake, insulin sensitivity and glycemic status in men and women. Am. J. Clin. Nutr..

[39] Juanola-Falgarona M, Salas-Salvado J, Monica B (2013). Association between dietary phylloquinone intake and peripheral metabolic risk markers related to insulin resistance and diabetes in elderly subjects at high cardiovascular risk. Cardiovasc. Diabetol..

[40] Dalmeijer GW, van der Schouw YT, Magdeleyns EJ (2013). Matrix gla protein species and risk of cardiovascular events in type 2 diabetic patients. Diabetes Care.

[41] Rasekhi H (2015). The effect of vitamin K1 supplementation on sensitivity and insulin resistance via osteocalcin in prediabetic women: a double-blind randomized controlled clinical trial. Eur. J. Clin. Nutr..

[42] Rasekhi H (2015). Phylloquinone supplementation improves glycemic status independent of the effects of adiponectin levels in premonopause women with prediabetes: a double-blind randomized controlled clinical trial. J. Diabetes Metab. Disord..

[43] Ibarrola-Jurado N, Salas-Salvadó J, Martínez-González MA, Bulló M (2012). Dietary phylloquinone intake and risk of type 2 diabetes in elderly subjects at high risk of cardiovascular disease. Am. J. Clin. Nutr..

[44] Beulens JW (2010). Dietary phylloquinone and menaquinones intakes and risk of type 2 diabetes. Diabetes Care.

[45] Ho Hsin-Jung, Shirakawa Hitoshi, Hirahara Keisukei, Sone Hideyuki, Kamiyama Shin, Komai Michio (2019). Menaquinone-4 Amplified Glucose-Stimulated Insulin Secretion in Isolated Mouse Pancreatic Islets and INS-1 Rat Insulinoma Cells. International Journal of Molecular Sciences.

[46] Zwakenberg SR (2019). Circulating phylloquinone concentrations and risk of type 2 diabetes: a Mendelian randomization study. Diabetes..

[47] Varsha MK, Thiagarajan R, Manikandan R, Dhanasekaran G (2015). Vitamin K1 alleviates streptozotocin-induced type 1 diabetes by mitigating free radical stress, as well as inhibiting NF-κB activation and iNOS expression in rat pancreas. Nutrition.

[48] Nakano T, Petrash JM (1996). Kinetic and spectroscopic evidence for active site of human aldose reductase. Biochem..

[49] El-Kabbani O (1994). Structures of human and porcine aldehyde reductase: an enzyme implicated in diabetic complications. Acta. Crystallogr. D. Biol. Crystallogr..

[50] Cousido-Siah A (2014). Identification of a novel polyfluorinated compound as a lead to inhibit the human enzymes aldose reductase and AKR1B10: structure determination of both ternary complexes and implications for drug design. Acta. Crystallogr. D. Biol. Crystallogr..

[51] Jorgensen WL (2000). Prediction of drug solubility from Monte Carlo simulations. Bioorg. Med. Chem. Lett..

[52] DeLano, W. L. The PyMOL Molecular Graphics System, DeLano Scientific, Palo Alto, Calif, USA, http://www.pymol.org (2002).

[53] Maggiora, G. M. & Johnson, M. A. Concepts and applications of molecular similarity; John Wiley & Sons: New York, pp 99– 117. (1990).

[54] Shelley JC (2007). Epik: a software program for pKa prediction and protonation state generation for drug-like molecules. J. Comp. Aided. Mol. Des..

[55] Friesner RA (2004). Glide: a new approach for rapid, accurate docking and scoring. 1. Method and assessment of docking accuracy. J. Med. Chem..

[56] Fanfrlik J (2013). Modulation of aldose reductase inhibition by halogen bond tuning. ACS Chem. Biol..

[57] Friesner RA (2006). Extra precision glide: docking and scoring incorporating a model of hydrophobic enclosure for protein-ligand complexes. J. Med. Chem..

[58] Abraham MJ (2015). GROMACS: High performance molecular simulations through multi-level parallelism from laptops to supercomputers. SoftwareX..

[59] MacKerell AD, Banavali N, Foloppe N (2000). Development and current status of the CHARMM force field for nucleic acids. Biopolymers..

[60] Zoete V, Cuendet MA, Grosdidier A, Michielin O (2011). SwissParam: a fast force field generation tool for small organic molecules. J. Comput. Chem..

[61] Leontyev IV, Stuchebrukhov AA (2012). Polarizable mean-field model of water for biological simulations with AMBER and CHARMM forcefields. J. Chem. Theory Comput..

[62] Tompa DR, Kadhirvel S (2018). Molecular dynamics of a far positioned SOD1 mutant V14M reveals pathogenic misfolding behavior. J. Biomol. Struct. Dyn..

[63] Yoshifumi F, Daisuke M, Haruki N (2009). Protein-ligand binding free energy calculation by the smooth reaction path generation (SRPG) method. J. Chem. Inf. Model..

[64] Lowry OH, Roserbrough NJ, Farr AL, Randall RJ (1951). Protein measurement with the folin phenol reagent. J. Biol. Chem..

[65] Ido Y (2010). Early neural and vascular dysfunctions in diabetic rats are largely sequelae of increased sorbitol oxidation. Antioxid. Redox Signal..

[66] Mylari BL (1991). Novel, potent aldose reductase inhibitors: 3,4-dihydro-4-oxo-3-[[5-(trifluoromethyl)-2-benzothiazolyl] methyl]-1-phthalazineacetic acid (zopolrestat) and congeners. J. Med. Chem..

[67] Burg M (1988). Role of aldose reductase and sorbitol in maintaining the medullary intracellular milieu. Kidney. Int..

[68] Warren JC, Murdock GL, Ma Y, Goodman SR, Zimmer WE (1993). Molecular cloning of testicular 20 alpha-hydroxysteroid dehydrogenase: identity with aldose reductase. Biochemistry..

[69] Vander Jagt DL, Robinson B, Taylor KK, Hunsaker LA (1992). Reduction of trioses by NADPH-dependent aldo-keto reductases. Aldose reductase, methylglyoxal, and diabetic complications. J. Biol. Chem..

[70] Kolb NS, Hunsaker LA, Vander Jagt DL (1994). Aldose reductase-catalyzed reduction of acrolein: implications in cyclophosphamide toxicity. Mol. Pharmacol..

[71] Kador PF, Wyman M, Oates PJ (2016). Aldose reductase, ocular diabetic complications and the development of topical Kinostat®. Prog. Retin. Eye Res..

[72] Ramasamy R, Goldberg IJ (2010). Aldose reductase and cardiovascular diseases, creating human-like diabetic complications in an experimental model. Circ. Res..

[73] Lee YWA, Chung SMS (1999). Contributions of polyol pathway to oxidative stress in diabetic cataract. FASEB J..

[74] Lee AY, Chung SK, Chung SS (1995). Demonstration that polyol accumulation is responsible for diabetic cataract by the use of transgenic mice expressing the aldose reductase gene in the lens. Proc. Natl. Acad. Sci. USA.

[75] Reddy AB (2011). Aldose reductase deficiency protects sugar-induced lens opacification in rats. Chem. Biol. Interact..

[76] Yeh LA, Ashton MA (1990). The increase in lipid per-oxidation in diabetic rat lens can be reversed by oral sorbinil. Metabolism..

[77] Srivastava SK, Ramana KV, Bhatnagar A (2005). Role of aldose reductase and oxidative damage in diabetes and the consequent potential for therapeutic options. Endocr. Rev..

[78] Oates PJ, Mylari BL (1999). Aldose reductase inhibitors: therapeutic implications for diabetic complications. Expert. Opin. Invest. Drugs..

[79] Engerman RL, Kern TS (1993). Aldose reductase inhibition fails to prevent retinopathy in diabetic and galactosemic dogs. Diabetes..

[80] Zheng X (2012). Partial inhibition of aldose reductase by nitazoxanide and its molecular basis. Chem. Med. Chem..

[81] Li HM, Hwang SH, Kang BG, Hong JS, Lim SS (2014). Inhibitory effects of Colocasia esculents (L.) Schott constituents on aldose reductase. Molecules..

