# Supplementary material for: Vitamin K1 prevents diabetic cataract by inhibiting lens aldose reductase 2 (ALR2) activity
Source: Sci Rep. 2019 Oct 11;9:14684. doi: 10.1038/s41598-019-51059-2 (PMC6789135; doi:10.1038/s41598-019-51059-2)
Supplement: Supplementary file 1 — Vitamin K1 prevents diabetic cataract by inhibiting lens aldose reductase 2 (ALR2) activity [file 41598_2019_51059_MOESM1_ESM.docx]

**Vitamin K1 prevents diabetic cataract by inhibiting lens aldose reductase 2 (ALR2) activity**

R. Thiagarajan^1*^, M.K.N. Sai Varsha^2^, V. Srinivasan^3^, R. Ravichandran^4^, K. Saraboji^1^

^1^School of Chemical & Biotechnology, SASTRA University, Tamil Nadu; ^2^Department of Biotechnology, Indian Institute of Technology, Madras, Chennai 600036; ^3^Disease Program Lead – Diabetes, MedGenome Inc., Bangalore, India; ^4^Diabetes Research Program, Division of Endocrinology, Department of Medicine, NYU Langone Medical Center, NY, New York 10016, USA.

*Correspondence to: [thiagi2007@gmail.com](mailto:thiagi2007@gmail.com)

**Current affiliation:** Department of Advanced Zoology & Biotechnology, Ramakrishna Mission Vivekananda College, Mylapore, Chennai 600004

Supplementary Figure S1: The molecular dynamics simulations indicate that the binding mode of vitamin K1 is consistent in the protein binding site. Structural superposition shows the closeup view on the binding site conformations of (a) ALR2 and (b) ALR1 molecules (carton representation) obtained during 50ns MD simulations. The conformations of vitamin K1 (sticks representation) observed at 5, 10, 15, 20, 25, 30, 35, 40, 45 and 50 ns time intervals are shown in cyan, magenta, yellow, salmon, gray, blue, red, green, white and red colours, respectively.

Supplementary Figure S2: The fluctuation of protein-ligand binding free energy (ΔG) values shows that the binding strength of vitamin K1 with ALR1 (red line) and ALR2 (black line) is comparable during 50ns MD simulation. Further, the binding of vitamin K1 is found stronger with ALR2 comparable to ALR1. The binding free energy (ΔG) values are calculated, for the conformations obtained in the MD traectory, using software myPresto version 5 (https://www.mypresto5.jp/en/).

Supplementary Figure S3: The binding sites of vitamin K1 in ALR1 (cyan sticks) and ALR2 (orange sticks) are located adjacent to NADPH binding site (white sticks); the glyceraldehyde molecule in its binding site is shown in yellow sticks, which is not in overlap with NADPH site but locates in the middle of vitamin K1 binding site. The ALR2 molecule is shown in surface representaiton for the overall perspective of binding mode.
